# Characterization of Polyester Nanocomposites Reinforced with Conifer Fiber Cellulose Nanocrystals

**DOI:** 10.3390/polym12122838

**Published:** 2020-11-28

**Authors:** Grazielle da Silva Maradini, Michel Picanço Oliveira, Gabriel Madeira da Silva Guanaes, Gabriel Zuqui Passamani, Lilian Gasparelli Carreira, Walter Torezani Neto Boschetti, Sergio Neves Monteiro, Artur Camposo Pereira, Bárbara Ferreira de Oliveira

**Affiliations:** 1 Forest and Wood Sciences Department, Federal University of Espírito Santo, Jeronimo Monteiro 29550-000, Brazil; graziellemaradini@gmail.com (G.d.S.M.); michelpicanco@gmail.com (M.P.O.); gabrielmguanaes@outlook.com (G.M.d.S.G.); gabriel.passamani@edu.ufes.br (G.Z.P.); lcarreira83@gmail.com (L.G.C.); 2 Forestry Engineering Department, Federal University of Viçosa, Viçosa 36570-900, Brazil; walterboschetti@gmail.com; 3 Military Institute of Engineering—IME, Materials Science Program, Praça General Tibúrcio 80, Urca, Rio de Janeiro 22290-270, Brazil; camposo.artur@gmail.com; 4 Advanced Materials Department, Northern Fluminense State University, Av Alberto Lamego, 2000, Campos dos Goytacazes 28013-602, Brazil; barbara.fo@gmail.com

**Keywords:** nanocomposite, mechanical behavior, thermal analysis, polyester, cellulose nanocrystal

## Abstract

The application of cellulose nanocrystal has lately been investigated as polymer composites reinforcement owing to favorable characteristics of biodegradability and cost effectiveness as well as superior mechanical properties. In the present work novel nanocomposites of unsaturated polyester matrix reinforced with low amount of 1, 2, and 3 wt% of cellulose nanocrystals obtained from conifer fiber (CNC) were characterized. The polyester matrix and nanocomposites were investigated by scanning electron microscopy (SEM), X-ray diffraction (XRD), bending test, and thermogravimetric analysis (TGA). The result showed that the addition of only 2 wt% CNC increased the nanocomposite flexural strength by 159%, the ductility by 500% and the toughness by 1420%. Fracture analyses by SEM revealed a uniform participation of the CNC in the polyester microstructure. The resistance to thermal degradation of the CNC reinforced nanocomposites was improved in more than 20 °C as compared to neat polyester. No significant changes were detected in the water absorptions and XRD pattern of the neat polyester with incorporations up to 3 wt% CNC. These results reveal that the 2 wt% CNC nanocomposite might be a promising more ductile, lightweight and cost-effective substitute for conventional glass fiber composites in engineering applications.

## 1. Introduction

The development of novel materials with improved properties to replace conventional materials has for a long time been the focus of attention of engineers and scientists [1]. In particular, the interest in polymer nanocomposites has recently been increasing [2]. Polymer composites are widely used materials due to their low cost, facility of processing, and applicability in new products. Particularly, in recent decades, many researches have been carried out to develop polymer nanocomposites and much attention has been focused on the addition of natural fiber nanocellulose as a reinforcement phase. This is mainly due to the current interest and need for development of sustainable products [1,3]. Cellulose nanocrystals show competitive advantages over synthetic industrialized materials, commonly used today as composites reinforcement, as they are renewable and biodegradable [3]. Moreover, cellulose nanocrystals have excellent properties such as low density, high crystallinity and improved physical and mechanical performance, associated with high Young’s modulus, high strength and low thermal expansion coefficient. In addition, abundance and low cost allow potential applications of nanocomposite materials [4,5]. These properties are described in the work of Movva and Kommineni [6], in which cellulose was extracted from pistachio peel and treated by acid hydrolysis, increasing the crystallinity index by 26%. This increase in crystallinity occurs because the acid hydrolysis destroys the amorphous regions present in the structure, leaving only the crystalline zones, which increased the cellulose Young’s modulus. This modulus of crystalline cellulose, depending on the method used for its determination, varies between 100 and 170 GPa, proving to be much higher than the elastic modulus of commonly used engineering materials [7].

Korotkov et al. [8] calculated the crystallinity index (CI) of different plant fibers microcrystalline and nanocrystalline cellulose obtaining 63% and 71%, respectively, which reveals their high CI. This can be explained because, with the extraction of cellulose on a nanometric scale, most of the defects associated with the hierarchical structure of the cellulose macromolecule are removed [9]. When applied as reinforcement in nanocomposites, due to the higher intrinsic performance of the nanometric filler, it is expected that cellulose nanocrystals improve the mechanical properties of the matrix as compared to cellulose fibers of macrometric size [7]. To obtain the aimed mechanical and thermal properties, compatible resins and reinforcements must be chosen, since the success in obtaining the nanocomposite depends on the properties of the filler, its dispersion in the polymer matrix and the interaction between the nanometric material and the polymer [1].

Among the thermoset polymer matrices used in the development of composite materials, polyester has advantages such as low cost, dimensional stability, and low cure temperature that allow it to be used in simplified molds [10]. Kargarzadeh et al. [11] and Zaghloul et al. [12] showed that the use of kenaf bast fiber cellulose nanocrystals as reinforcement in unsaturated polyester matrix has generated nanocomposites with excellent mechanical properties. Kargarzadeh et al. [11] added 2% of silane-treated kenaf cellulose nanocrystals to the polyester matrix and observed that the tensile strength was improved by 20% in comparison to neat polyester without reinforcement. There was also an improvement of 10% in the elastic modulus of the nanocomposite, which the authors attribute to the high stiffness of the cellulose nanocrystals. Zaghloul et al. [12] achieved an improvement in tensile strength of 30.5% by adding 4% of cellulose nanocrystals in polyester nanocomposite.

The hypothesis raised in the present study is that the addition of conifer cellulose nanocrystals (CNC) in low proportions to polyester nanocomposites, using styrene monomer as a compatibilizing agent between the matrix and the reinforcement filler, would contribute to enhance the mechanical and thermal properties of the nanocomposite. Hence, the aim of this research is to investigate the incorporation of different concentrations of cellulose nanocrystals obtained from conifer fiber in an unsaturated polyester matrix in order to improve the physical, chemical, mechanical, and thermal properties of nanocomposites. Another objective is to verify if the present novel nanocomposites were developed with improved mechanical and thermal performance, using a low cost and easy application-compatibilizing agent.

## 2. Materials and Methods 

### 2.1. Materials

**Figure 1:**
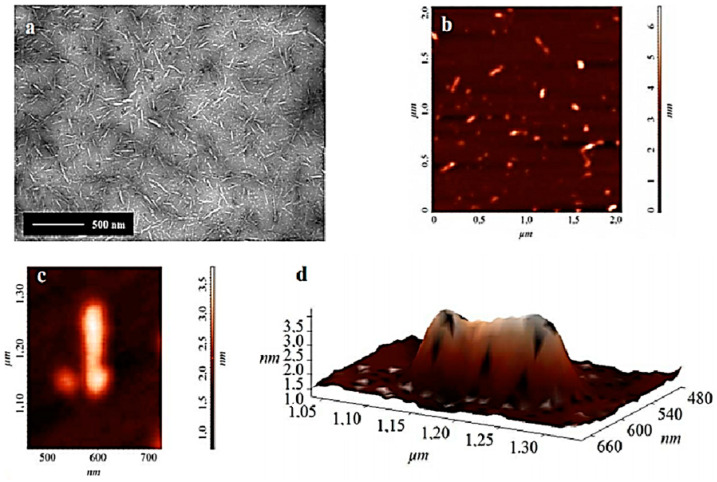
(**a**) TEM of the commercial conifer cellulose nanocrystals (CNC); (**b**) AFM, 2 × 2 μm scan; (**c**) Scan of 300 × 400 nm (detail of a nanocrystal), (**d**) 3D image of the surface of the commercial nanocrystal.

Commercial conifer fiber cellulose nanocrystals (CNC) were supplied by the Development Center of the University of Maine, Orono, (USA). Figure 1 shows transmission electrons microscopy (TEM) and atomic force microscopy (AFM) images as well as details and 3D topographic surface of conifer commercial CNC. Average CNC dimensions obtained using the Image-Pro Plus software were found as 190 ± 15 nm length and diameter of 3.0 ± 0.5 nm.

Polyester resin (UC 2120 AC PLUS), indicated with molecular weight of M_n_ = 9 × 10^3^ g/mol, as well as butanox catalyst (M-50) styrene monomer solvent, and white silicone rubber (PS) were all supplied by Redelease (Brazil).

### 2.2. Processing of Nanocomposites 

The CNCs were dispersed in the styrene monomer and added to the polyester resin in different percentages. The materials were homogenized for approximately 5 min. The specimens were produced with the shapes and dimensions as per ASTM D7264 standard [13]. 

The composites were developed with the addition of 1, 2, and 3 wt% of cellulose nanocrystals in the polyester matrix. For all concentrations, 28 wt% styrene monomer was added in the mixture to disperse the CNCs. The choice for these concentrations was due to preliminary laboratory tests proving that higher percentages of reinforcement load no longer guaranteed the mechanical increase, by facilitating the occurrence of nanocrystal agglomerations. The amount of polyester resin required for the production of four specimens was calculated for each filler/matrix concentration. The CNC was added into the still fluid polyester resin and thoroughly mixed according to the desired calculated percentages. The amount of catalyst addition was that recommended by the manufacturer to be 1 wt%. The mixture was poured into a 15 mm thick silicone mold and, after 24 h of curing, the complete polymerization occurred. 

### 2.3. X-ray Diffraction 

X-ray diffraction (XRD) measurements were performed using a Rigaku MINIFLEX—600 diffractometer (Tokyo, Japan). The samples were scanned in the range (2θ) from 4° to 70°, at a scan rate of 2 °/min and a step of 0.02°.

The crystallinity index (CI) of the CNC was calculated using the empirical method proposed by Segal et al. [14] according to the equation:

(1) $$CI=\frac{I_{\left(200\right)}\;-\;I_{\left(am\right)}}{I_{\left(200\right)}}$$

in which *I*_(200)_ is the maximum intensity of the main diffraction peak related to the crystalline plane, with diffraction at 2θ = 22° and 2θ = 20° and *I*_am_ refers to the amorphous halo at 2θ = 18° and 2θ = 16° for cellulose I and cellulose II, respectively [15,16].

The *CI* of the neat polyester and nanocomposites reinforced with 1, 2, and 3 wt% CNC was calculated using the Match 3 software.

### 2.4. Scanning Electron Microscopy (SEM)

The fracture region of the samples was analyzed using the model SSX-550 Shimadzu scanning electron microscope (Tokyo, Japan). The samples were previously fixed on a metallic support with carbon tape and then metallized with gold, to guarantee the precise scanning of the secondary electrons on the sample during the microscopic analysis. The images were obtained at 10 kV.

### 2.5. Bending Tests

**Figure 2:**
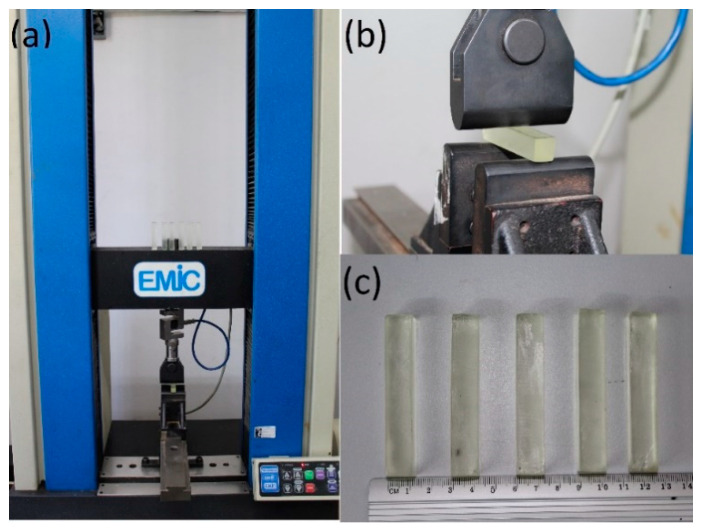
Experimental procedure of bending test, (**a**) universal testing machine, (**b**) setup support, and (**c**) standard specimens.

For the structural evaluation of the developed composites strength, three-points bend tests were performed on five specimens for each composition (Section 2.1). The dimensions of the bend test specimens were 70 mm in length, 13 mm wide, and 13 mm thick according to the ASTM D7264 standard [13]. The support span was set to 60 mm. To perform the bending tests, a model DL 10000 EMIC universal testing machine (São José dos Pinhais, Brazil), shown in Figure 2, was used. All the specimens were tested at a crosshead speed of 1 mm/min until the specimen break. The calculation of the optimal proportion of CNC in the matrix was performed by values obtained from polynomial adjustment. The flexural strain and flexural toughness were calculated according to a methodology presented elsewhere [17,18]. Figure 2 illustrates the experimental setup and specimens used in the bending test.

### 2.6. Thermal Analyses

The resistance to thermal degradation of the samples was studied by thermogravimetric analysis (TGA), and differential thermal analysis (DTA). The model Labsys EVO Setaran thermal analyzer (Caluire, France) was used for all measurements. Samples with 30 mg were analyzed from room temperature (RT~25 °C) to 800 °C, at a heating rate of 10 °C min^−1^, under a nitrogen atmosphere. 

### 2.7. Walter Absorption Test

Water absorption test was performed as per ASTM D570 [19] in order to investigate the behavior of the nanocomposites when subjected to moisture. An immersion procedure was applied for 24 h. Five samples of each composite with 1, 2, and 3 wt% of CNC as well as neat polyester specimens without CNC reinforcement (0 wt%), for control were tested. The dry samples were weighed on a four-digit analytical balance (with precision of 10^−4^ g). After initial weighing, the composites were immersed in distilled water at RT and maintained in this condition for 24 h. Following the immersion, the samples were cleaned with a dry cloth and weighed again. The percentage of water absorbed by the samples was calculated from the difference in weight ∆*W* between the wet sample and the dry sample, according to:

(2) $$\Delta W\left(\%\right)=\;\frac{W_{w}-W_{d}}{W_{d}}\;\times \;100$$

where *W_s_* and *W_d_* are the weight of the wet and dry sample, respectively. 

After another 24 h of immersion in distilled water, the samples were again dried and weighed, and then placed in a desiccator for 24 h. After this period, the composites were once more weighed. The percentage of nanocomposites soluble matter lost (*W_L_*) during immersion was calculated by the difference between the sample weights before immersion and after drying in the desiccator, according to:

(3) $$W_{L}\left(\%\right)=\;\frac{W_{S}-W_{R}}{W_{S}}$$

where *W*_S_ and *W_R_* are the weight of the sample before immersion and after drying in the desiccator, respectively. The percentage of water absorbed is the sum of the values obtained from Equations (2) and (3). 

In order to analyze the data, a completely randomized design was used, in which the data were tested for normality. The dependent variables are the CNC proportions over the polyester matrix and the analysis of variance (ANOVA) was performed. When considered significant, regression test was applied to obtain the models and analyze the estimate curves. 

## 3. Results and Discussion

### 3.1. X-ray Diffractometry 

**Figure 3:**
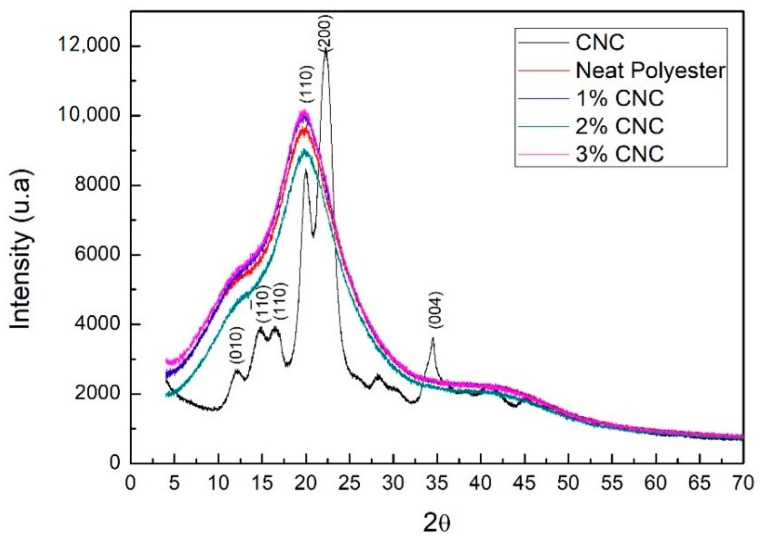
Comparative X-ray diffraction between CNC, polyester, and nanocomposites.

Figure 3 shows the XRD pattern obtained for CNC and polyester, as well as for the nanocomposites. In this figure, the CNC presents a well-defined mixture of cellulose I and cellulose II polymorphs. The presence of cellulose type II is observed by characteristic peaks at 2θ = 12°, 20° and 22° and cellulose type I due to the presence of peaks at 2θ = 14.5°; 17.5°; 22.0° and 34.6° [15,20,21,22]. This indicates that after the alkaline pre-treatment of cellulose, used before acid hydrolysis to obtain the CNC, the native cellulose (type I) became cellulose II, which presents a more stable structure [15].

The CNC crystallinity index (CI), calculated from Equation (1), was found as 76.5%. Other authors have reported the degree of crystallinity in the range of 70 to 90% for nanocrystals isolated from different sources by acid hydrolysis [8,15,23]. It is suggested that the present high CI indicates the effectiveness of acid hydrolysis and the increased rigidity of the crystalline cellulose [6]. The crystallinity of cellulose is an important factor in determining its reinforcing capacity and its mechanical and thermal resistance in applications for the development of composite materials [24]. Crystalline nanocrystals are expected to generate a more effective reinforcement in composites, due to increased stiffness, reaching a higher Young’s modulus [15,25,26]. The polyester XRD pattern in Figure 2 reveals two halos. The main and most accentuated one, at approximately 19.75°, is characteristic for amorphous and semi-crystalline materials [27]. The second halo occurred at approximately 41.36°. The diffraction patterns of the nanocomposites are close to that of the polyester, with no characteristic peak formation. However, there is a slight change in the intensity of the nanocomposites diffraction peaks compared to the polyester. This result is in agreement with that obtained by del Pino et al. [28], in which the XRD pattern of polyester composites reinforced with curaua fibers and organophilic clay nanoparticles presented a behavior similar to that of the polyester resin without reinforcement, with peaks at 2θ = 22° and 2θ = 43° displaying similar intensities. 

Table 1 shows the crystallinity indexes of the polymer without reinforcement and nanocomposites crystallinity indexes reinforced with 1, 2, and 3 wt% of CNC. Analyzing the CI values of the nanocomposites shown this table, there is evident occurrence of interactions between the cellulose nanocrystals and the polymer matrix. Indeed, there are variations in the CI of the nanocomposites in comparison with that of the polyester without reinforcement. It is possible to notice that this interaction was enhanced for nanocomposites reinforced with 1 and 2 wt% CNC, whereas in the nanocomposite with 3 wt% CNC the filler/matrix interaction, the reinforcement is comparatively decreased. It is believed that in nanocomposites with 1 and 2 wt% CNC, the reinforcement promoted nucleation sites of atomically ordered regions, which resulted in an increase in the IC of these materials [24]. On the other hand, with the addition of 3% CNC, excessive agglomeration of cellulose nanocrystals may have occurred, leading to the formation of voids, which generated defects in the materials, thus decreasing the crystallinity of the nanocomposite.

Liu et al. [27] added commercial cellulose microcrystals in concentrations of 20, 30, and 40 wt% in acrylated epoxidized soy oil resin. The authors observed characteristic XRD peaks of the two components, indicating the formation of crystalline regions in the composites, due to the addition of microcrystals. This corroborates the results obtained in the present work for the CNC.

### 3.2. Bending Test

**Figure 4:**
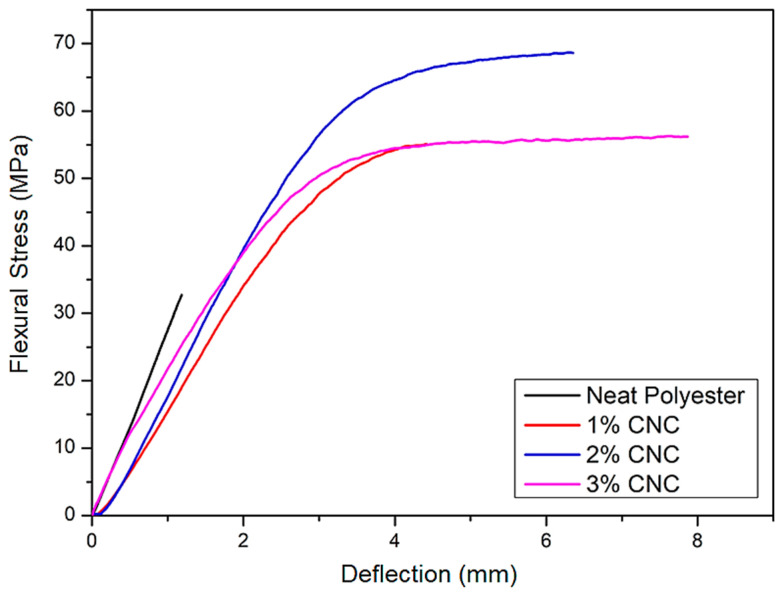
Typical flexural stress vs. deflection curves for the neat polyester and nanocomposites reinforced with CNC.

Figure 4 shows typical flexural stress versus deflection curves for the neat polyester and CNC reinforced polyester nanocomposites. In these curves, it should be noticed the brittle behavior of the polyester with practically no plastic deformation. In contrast, all nanocomposites display significant plastic region with comparatively increased total deflection and higher flexural strength at maximum stress. 

**Figure 5:**
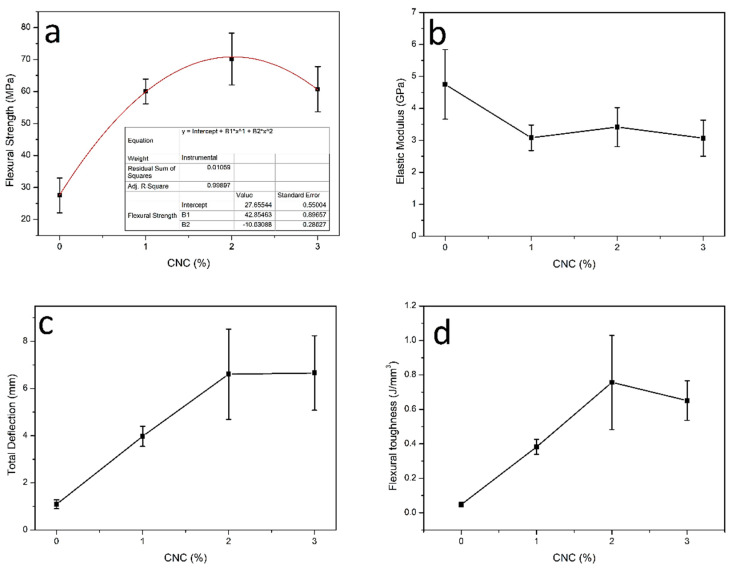
Variation with weight percent CNC (wt%) of: (**a**) Flexural strength, (**b**) elastic modulus, (**c**) total deflection, and (**d**) flexural toughness.

The curves in Figure 4 reveal that the incorporation of CNC in the polyester matrix improve the mechanical bending properties of the nanocomposites. Based on all curves, such as those in Figure 4, main bending properties are graphically shown in Figure 5. 

It can be observed in Figure 5 that the composites reinforced with different amount of CNC present results of flexural strength, total deflection (strain) and toughness superior to the corresponding values obtained with the neat polyester. As for the elastic modulus, within the standard deviations, one may consider that the incorporation of CNC does not practically affect the nanocomposite stiffness. The nanocomposites mechanical properties are directly related to the microstructural parameters such as the dispersion of nanocrystals in the polymer matrix and the matrix/nanocrystal interfacial bond [28]. From the results in Figure 5, it can be suggested that there is a good dispersion of the CNC in the polyester matrix for the investigated concentrations, as well as a favorable matrix/nanocrystal interaction, as indicated by the further discussed SEM analysis. This good interaction between the cellulose nanocrystals and the polymer matrix might be associated with the addition of styrene monomer in the mixture, which helped the dispersion of the filler in the matrix. 

Figure 5a shows that the addition of 1, 2, and 3 wt% CNC in the polyester matrix provided an increase in flexural strength of, 122%, 159%, and 122%, respectively. ANOVA and Tukey’s test, Table 2 and Table 3, respectively, reveal that there is no significant difference between the flexural strength values of composites with 1, 2, and 3 wt% CNC. However, they differed significantly from the neat polyester, which proves that the addition of these contents, as reinforcement load, improved the nanocomposite mechanical strength. Considering the polynomial adjustment (y = 27.7 + 42.9x − 10.6x^2^) in Figure 5a, it is noticed that the CNC optimal concentration in the polymer matrix is 2 wt%. Higher concentrations of CNC can cause agglomerations of nanocrystals and, consequently, heterogeneous dispersion in the polyester matrix, which reduces the effective reinforcement. This fact is demonstrated by the work of Rehman et al. [29], in which the addition of 7% cellulose microcrystals to epoxy composites with alkali-treated jute fibers resulted in a 52.14% increase in the flexural strength. However, an additional increase in the content of cellulose microparticles decreased the flexural properties of the composites. 

Studies carried out by Johar and Ahmad [30] and Zaghloul et al. [12] also corroborate the results obtained in the present study, since their tensile strength increased with the addition of rice cellulose nanocrystals to both starch and polyester matrices, respectively. However, when adding high concentrations of cellulose nanocrystals to the matrix, namely 8 wt% [29] and 6 wt% [12], the mechanical strength of the materials decreased. Indeed, due to the cellulose being in a nanometric dimension, there is a tendency to form larger agglomerates when its amount is increased. This phenomenon results in a strong self-interaction between nanocrystals and, consequently, a reduced interaction between them and the matrix [30]. The formation of nanocrystals agglomerates cause defects in the material, as they act as stress concentrators, resulting in a lower mechanical resistance of polyester nanocomposites reinforced with cellulose nanocrystals [12,31].

Shojaeiarani et al. [32] observed that nanocomposites developed with non-chemically treated wood cellulose nanocrystals showed a weak interaction with the matrix, allowing the formation of relatively large aggregates of nanocrystals, making it difficult to homogeneously disperse the reinforcing agent in the polymer. Thus, the incorporation of untreated cellulose nanocrystals in the matrix might not result in a significant change in the mechanical properties of the nanocomposites.

It is noteworthy that in the present work no chemical treatment was carried out on the use CNCs. Indeed, based on the results obtained, an increase in the flexural strength and toughness of the composites in relation to the neat polyester without reinforcement was observed. This indicates a good interaction between the nanocrystals of cellulose and the polyester matrix, which may be related to the addition of styrene monomer in the preparation of composites, since this solvent reacts chemically with the unsaturated polyester and helps in the dispersion of the reinforcement CNC in the matrix.

Figure 5b shows that the addition of 1, 2, and 3 wt% of CNC to the polyester, within the standard deviations, caused practically no change to the elastic modulus. Thus, the increase of CNC in the polyester matrix did not affect the stiffness. The cellulose was crystalline and, in general, greater crystallinity means stiffer composites [33,34,35]. Asadi et al. [34] discussed that a more rigid interface results in a more effective load transfer through the fiber/matrix interface and, consequently, in a higher elastic modulus for the composite. However, in the present case the low amount of CNC was not enough to change the nanocomposites stiffness. In fact, ANOVA and Tukey’s test, Table 4 and Table 5, respectively, did not show significant difference between the nanocomposite flexural modulus and that of the polyester without reinforcement. In addition, there was no significant difference between all nanocomposites.

Figure 5c shows that the total deflection, associated with the flexural strength, of the nanocomposites was significantly increased in comparison to the neat polyester. Indeed, CNC incorporation of 1, 2, and 3 wt% raised the bending deflection by 263%, 500%, and 609%, respectively. This was mainly a consequence of plastic deformation developed by the nanocomposites. These results highlight the interaction potential of CNC with the polyester matrix, which is also capable of improving the nanocomposites’ ductility.

By means of the ANOVA parameters presented in Table 6, the hypothesis that total deflection values are equal ought to be rejected with a 95% level of confidence since F_cal_ is higher than F_tab_. Moreover, the Tukey’s test honesty significant difference in Table 7 proves that the 2 wt% CNC nanocomposite has the best total deflection (strain) associated with plastic deformation.

As for the flexural toughness, Figure 5d clearly indicates that the addition of CNC is responsible for a higher amount of absorbed energy. Similar results have recently been reported by Neuba et al. [36] for the tensile toughness of epoxy composites reinforced with natural lignocellulosic fibers. Luz et al. [37] demonstrated that many other lignocellulosic fibers contribute to reinforced several polymer matrix composites. Based on the results in Figure 5d, the addition of 1, 2, and 3% wt% CNC in the polyester matrix causes an increase in flexural toughness of 660%, 1420%, and 1200%, respectively.

Table 8 and Table 9 present the ANOVA and Tukey test parameters for the corresponding data of flexural toughness in Figure 5d. Based on these parameters, the hypothesis that the flexural toughness results are equal should be rejected with 95% level of confidence. Moreover, the Turkey’s test indicates that the 2 wt% CNC nanocomposite has the highest toughness.

The flexural results shown in Figure 5 for polyester nanocomposites reinforced with relatively low, 2 wt%, of CNC demonstrate a promising engineering material. Indeed, with flexural strength of 70 MPa and toughness of 0.76 J/mm^3^, the relatively low density, cost effective and more ductile 2 wt% CNC nanocomposite would compete with commonly applied glass fiber/polyester composite [38]. Low amounts of reinforcement have been shown to be effective in other composites used as engineering material. Lu et al. [39] reported that 2 wt% PVA addition into cementitious composite improved by 23.5% the tensile strength.

### 3.3. Microstructural Evaluation of Nanocomposites by (SEM)

**Figure 6:**
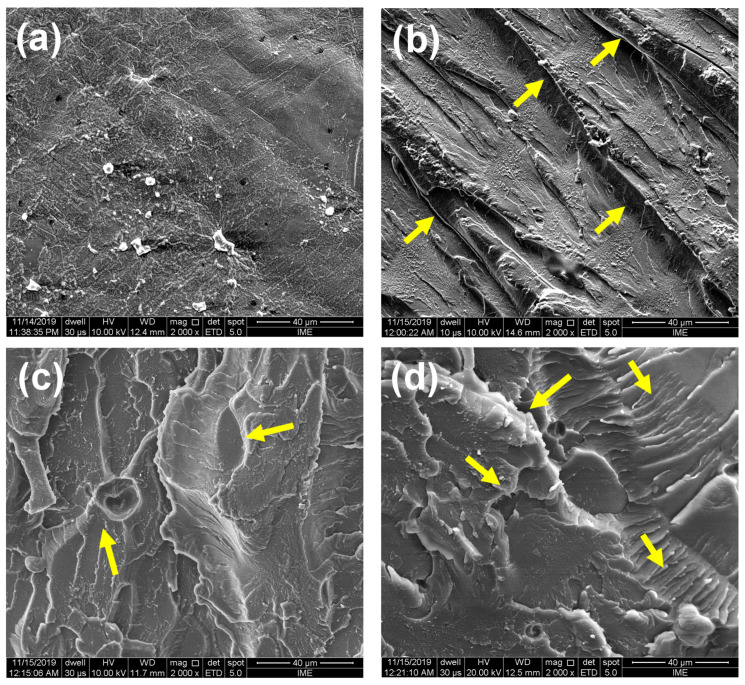
Scanning electron microscopy (SEM) images of (**a**) polyester as well as nanocomposites with (**b**) 1 wt% CNC, (**c**) 2 wt% CNC, and (**d**) 3 wt% CNC.

Figure 6 shows SEM images of the bending fractured surface of polyester composites reinforced with different CNC concentrations. In Figure 6a, the fractured surface of the polyester without reinforcement is smoother than those of the nanocomposites. The roughness in the composites, Figure 6b–d, indicated by the arrows pointed to ridges, characterizes the increased crack arrest caused by the addition of CNC and the styrene monomer to the matrix. This may also be attributed to the stress concentration due to the incorporation of CNC in the matrix. River marks in the right side of Figure 6d apparently indicate an accentuated effect, which might be assigned to agglomeration of nanocrystals in the 2 wt% CNC nanocomposite.

The presence of rigid CNC particles in the polyester contributes to the initiation of energy absorption mechanisms in association with the appearance of crack. These energy dissipation mechanisms induce more local plastic deformation, close to the crack tip [40]. Through the SEM micrographs shown in Figure 6, it was not possible to identify the CNC in the matrix. However, it reveals that the addition of these CNC particles in the polyester caused significant structural changes in the nanocomposites, such as roughness and undulations pointed by arrows.

When the load is applied to a fragile material like the polyester, numerous micro cracks are simultaneously formed. The distribution of these micro-cracks depends essentially on the concentration of local stress and the material heterogeneity. The formation of micro-cracks around the CNC reduces the stress concentration and interferes with further propagation of cracks. On the other hand, the presence of stress concentrators can lead to shearing around the particles over a large volume of the material and not just at the crack tip [40]. The roughness and undulations on surface of the nanocomposites suggest that the matrix presents an effective dispersion of the nanocrystals, since it was not possible to observe the occurrence of CNC agglomerates.

### 3.4. Thermogravimetric Analysis (TGA), Derivative Thermogravimetry (DTG), and Differential Thermal Analysis (DTA)

**Figure 7:**
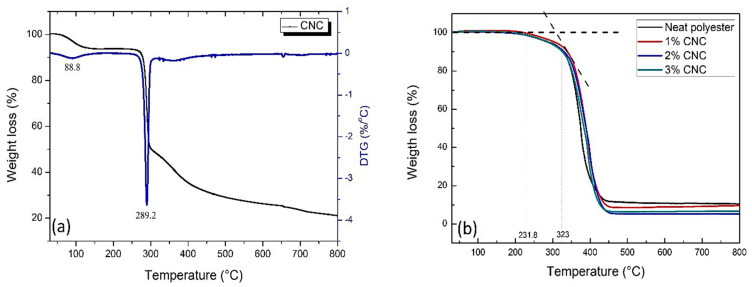
(**a**) Thermogram (TG) and its derivative (DTG) for CNC and (**b**) comparative thermogram of neat polyester and nanocomposites with different CNC concentrations.

Figure 7 shows the results obtained by thermogravimetric analysis carried out on CNC, neat polyester and nanocomposites reinforced with 1, 2, and 3 wt% CNC. In Figure 7a, an initial mass loss of the CNC is observed between 60 °C to approximately 120 °C, which can be attributed to release of moisture, since the CNC are hydrophilic and consequently absorb moisture easily. After the water loss event, up to 265 °C, it is shown that the CNC are thermally stable and only at higher temperatures effective thermal degradation occurred.

Borsoi et al. [16] studied the thermal degradation behavior of nanofibers and cellulose nanocrystals. The authors state that two main events were observed in the loss of cellulose mass. The first event occurred at temperatures from 60 to 110 °C, which was attributed to water loss and the second occurred around 300 °C was attributed to cellulose degradation and decomposition. Kakati et al. [41] reported that jute fabric/polyester composites exhibited similar thermal behavior with two stages of degradation. The first occurs at a lower temperature, between 40 and 120 °C, due to the vaporization of moisture present in the materials. The second and main degradation occurs at higher temperatures, between 230 and 530 °C, which the authors attribute to the decomposition of lignocellulosic fibers. Their values of cellulose and lignocellulosic fibers thermal degradation fibers are close to the results obtained in the present work for CNC thermal degradation.

Van de Velde and Kiekens [42] explain that below 300 °C, cellulose degradation reactions, which correspond to dehydration, are slow and the complete cellulose degradation occurs in an excessive period, taking days to complete. However, at temperatures above 300 °C the rapid degradation reaction takes place, where the break of hydrogen bonds usually occurs, causing changes in crystallinity, forming free radicals, carbonyl and carboxyl groups, which accelerates the degradation of primary cellulose. It is noteworthy in Figure 7a that a first DTG peak occurred at 88.8 °C, which corresponds to water release. The second and main peak occurred at approximately 289.2 °C, indicating that at this temperature the maximum rate of degradation of CNC occurred. Although the value here obtained is below the one observed in the literature, it is relatively close to the range of maximum rates reported for cellulose degradation that occurs between 310 and 390 °C [42]. In Figure 7b it is shown that thermal degradation of polyester and nanocomposites starts at a temperature close to 231.8 °C, and becomes more intense at approximately 323 °C. Up to 200 °C the variation in the material weight is very low, being insignificant. In a temperature range between 200 to 300 °C, the neat polyester loses only 5.49% of its mass, while polyester matrix composites reinforced with 1, 2, and 3 wt% of CNC lose 5.46%, 5.90%, and 5.98%, respectively of their initial mass. Therefore, both the polyester and the nanocomposites showed good thermal stability up to 300 °C, which might be considered the working temperature for practical application.

A study by Ferreira et al. [43] pointed out that polyester loses 2% of its weight at 175 °C, while the beginning of a great mass loss occurs at higher temperatures, around 330 °C. In this research, at temperatures above 323 °C the nanocomposites underwent the process of thermal degradation and their maximum rate occurred around to 400 °C. Kargarzadeh et al. [11] developed unsaturated polyester matrix composites reinforced with cellulose nanocrystals from kenaf fiber and investigated the weight loss with an increase in the temperature. Their TG thermograms showed that the decomposition of polyester and polymer composites reinforced with CNC, both treated with silane and without chemical treatment, occurred intensely at a temperature close to 300 °C, which corroborates the results obtained in the present study.

**Figure 8:**
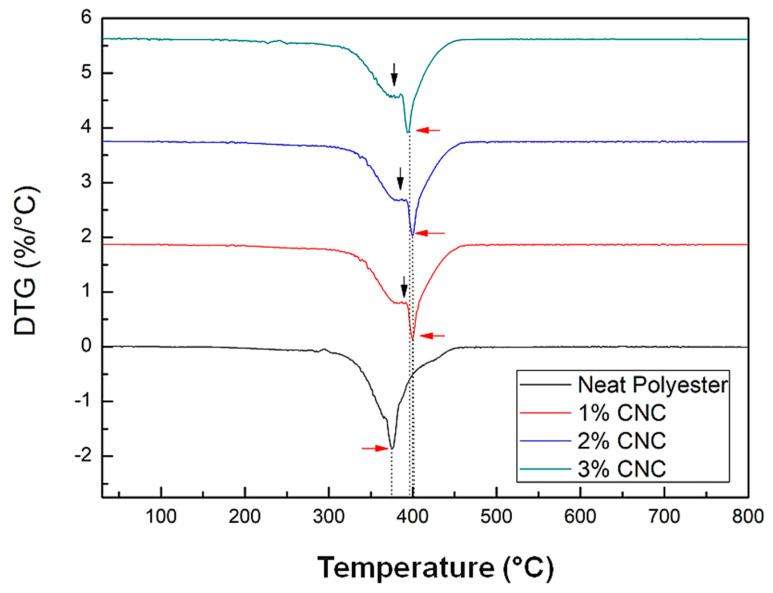
Comparative DTG between nanopolymers with different contents of commercial CNC.

Figure 8 depicts details of the neat polyester and nanocomposites DTG curves. In this figure, accentuated DTG shoulders for the nanocomposites at approximately 386 °C are indicated by vertical black arrows. These events correspond to secondary thermal reactions which correspond to the polyester DTG peak. Analyzing the nanocomposites main DTG peak, indicated by horizontal red arrows in Figure 8, it can be noticed that the neat polyester degraded at a lower temperature compared to those of the nanocomposites. In addition, the maximum rate of polyester thermal degradation was approximately 376 °C and those of the nanocomposites reinforced with 1, 2, and 3 wt% of CNC, occurred at approximately 400 °C.

Therefore, it might be concluded that the addition of up to 3 wt% of CNC in the unsaturated polyester matrix increased the resistance to thermal degradation of the material by approximately 20 °C. This indicates that, even without CNCs chemical treatments, there was a strong interaction between nanocrystals and the polymer matrix, improving the thermal stability of the developed nanocomposites. Thus, the use of styrene monomer as a compatibilizing agent between the reinforcement load and the matrix was effective, as this solvent contribute to dispersion and interaction between CNC and the matrix. This strong interaction was also confirmed by the flexural strength and toughness improvement in the nanocomposites disclosed in Figure 5.

The values obtained in this study are in agreement with those reported in the literature. Kargarzadeh et al. [11] showed that the DTG curve exhibited two peaks, one at 370 °C, corresponding to cellulose decomposition and the largest decomposition peak at 380 °C, attributed to the polyester. For composites with 2 and 4 wt% of kenaf cellulose nanocrystals, the peak remained at 380 °C, while for the composite with incorporation of 6 wt%, the peak occurred at 386 °C. The authors discuss that there was an improvement in the cellulose nanocrystals resistance to thermal degradation when incorporated into the polyester matrix. The main peak of DTG for the cellulose nanocrystals was around 300 °C and moved to 370 °C with the nanocrystals incorporation into the polyester resin. According to the authors, this improvement in cellulose nanocrystals thermal stability suggests that strong interactions occurred between the load and the matrix, especially after the treatment with silane.

Hence, in the present work, although the increase in thermal resistance is relatively low, the nanocomposites can be applied at temperatures higher than 20 °C of the corresponding resistance temperature of the unsaturated polyester. These results coincide with studies reported in the literature [5], in which the increment of cellulose nanocrystals in the polymer matrix increased the thermal stability of the matrix.

**Figure 9:**
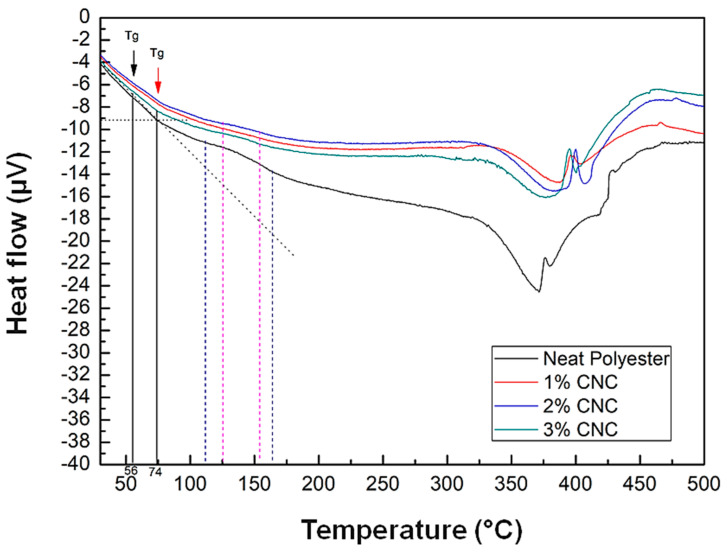
DTA curves for unsaturated polyester as well as nanocomposites with 1, 2, and 3 wt% of CNC. The black arrow indicates the polyester T_g_ and the red arrow indicates the composites T_g_.

Figure 9 illustrates the DTA curve of the studied samples. The downward peaks correspond to endothermic transitions, while the upward peaks correspond to exothermic events. In this figure, one sees that, although there are no marked transition decays, it is possible to observe that the polyester glass transition temperature (T_g_) occurred close to 56 °C, while those of the nanocomposites occurred close to 74 °C. This suggests that the increase in CNCs in the resin increased the resistance to the chains mobility in the polyester amorphous phase, causing the crystalline/amorphous transition of the material to occur at a temperature higher than that of the polyester without reinforcement [41]. The increase in T_g_ with the addition of CNC might be associated with the increase in the strength and toughness of the material, which was proven through the bending test and indicated by the SEM analysis. This phenomenon reinforces the strong interaction between the CNC and the polyester matrix, caused by the use of styrene monomer as a compatibilizing agent. This increase in T_g_ might also contribute to preserve the nanocomposites crystallinity indices up to 74 °C.

Kakati et al. [41] added 10 wt% of oil-based resin from *Ricinodendron heudelotii* to the polyester composite reinforced with jute fibers and observed that T_g_ increased from 72.6 to 126.8 °C. These results are in agreement with the results obtained in the present research, since with the addition of the CNC in the polyester matrix there was an increase in the T_g_ of the material.

Exothermic peaks are associated with the range of cure temperature of the material [44]. Figure 9 also presents, in association with exothermic peaks, the initial and final curing temperatures of the polyester and the nanocomposites in blue and purple vertical dashed lines, respectively. By observing these peaks, it can be noticed that the beginning of the polyester curing (blue) occurred around 110, while composites (purple) occurred at 125 °C. The cure reactions were completed at 163 °C for polyester and 154 °C for the nanocomposites. These values suggest that the curing reaction occurred more quickly in the composites compared to the neat polyester, indicating once again good interactions between the reinforcement and the matrix.

The results obtained in the present study differ slightly from the values reported in the literature. This occurs because the T_g_ value depends on the heating rate used during the analysis. The increase in the heating rate causes changes of T_g_ to a higher temperature [45].

### 3.5. Water Absorption

**Figure 10:**
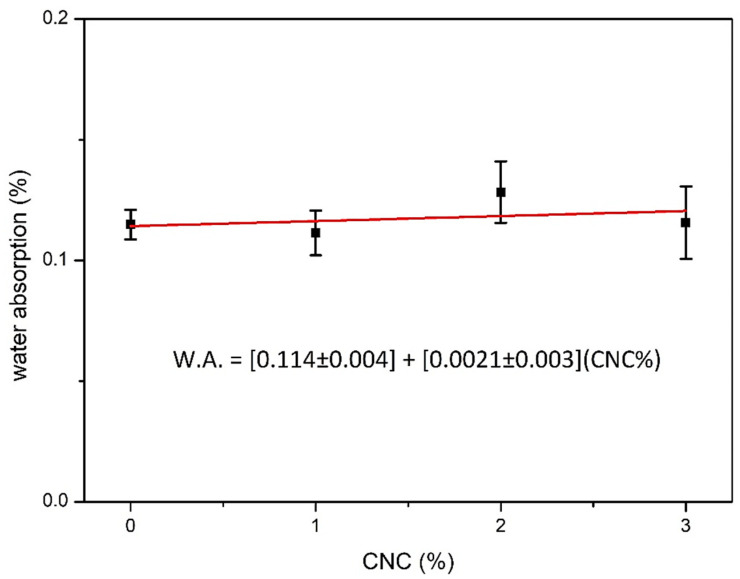
Comparative water absorption between unsaturated polyester resin polymer and nanocomposites reinforced with different CNC concentrations.

The great disadvantage in the use of cellulose nanocrystals as a reinforcement phase in polymer composites is due to its extreme sensitivity to water, which drastically reduces the mechanical performance of the material in a humid environment [11]. The hydrophilic nature of cellulose occurs due to the presence of hydroxyl groups in its structure, which establish hydrogen bonds with water molecules [46,47]. Thus, this study investigated the water absorption of the composites developed as a function of the CNC concentration in the unsaturated polyester, when immersed in water during a 24-h period. Figure 10 shows the water absorption of the neat polyester and nanocomposites reinforced with 1, 2, and 3 wt% CNC.

It can be noticed in Figure 10 that the composite with 2 wt% of CNC had a slightly higher water absorption when compared to the others. Thus, this result allows an interpretation that the concentrations of CNC added in the polyester did not demonstrate interference in the moisture resistance of the material after 24 h of immersion. This was confirmed by the ANOVA, in which the difference between the results of all treatments was not significant. Although the CNCs are hydrophilic, the concentrations added to the polyester resulted in a relatively low interference in the water absorption by the nanocomposites when subjected to moisture. Thus, these materials have a good resistance to moisture, when submerged for up to 24 h. Water absorption tests are being carried out in ongoing research for longer periods.

It is known that cellulose nanocrystals are formed by the crystalline domains of cellulose. Moudood et al. [47] explain that the water absorption of cellulose microfibers depends on their crystallinity. The authors mention that the volume of water absorbed by the cellulose decreases when the crystallinity rate increases and the diffusion of moisture in the cellulose occurs mainly in the amorphous phase. Therefore, although CNCs are hydrophilic, their water absorption is lower when compared to the water absorption by the cellulose macromolecule. The high crystallinity of the CNC applied in this research, confirmed by the XRD analysis, helped in the low water absorption of the nanocomposites with up to 3 wt% CNC. Moreover, Liu et al. [27] explain that low levels of cellulose microcrystals present in composites can be completely covered by the resin, which prevents direct contact with water molecules, making it difficult to absorb moisture [48]. Indeed, in polymer composites reinforced with natural fibers, water absorption depends on the diffusion of water molecules through micro-gaps in the polymer chain and on the interface between the fibers and the matrix [46,49].

The low water absorption by the nanocomposites corroborate the fact that there was a good interaction between the CNC and the polyester matrix. This fact is also evidenced by the flexural results and thermal analysis, revealing that up to 2 wt%, no CNC agglomerations might have occurred. Owing to the good dispersion of 1 an 2 wt% CNC in the polyester matrix, there was no micro-void formation during the polymer cross-linking process, as demonstrated by the samples fracture region photomicrographs in Figure 6, not allowing the water molecules to penetrate into the matrix [11].

## 4. Conclusions

The incorporation of cellulose nanocrystals (CNC) with size around 3 nm in diameter, in an unsaturated polyester matrix resulted in significant improvement in mechanical flexural properties, thermal behavior and water absorption, despite the low amounts of 1, 2, and 3 wt% of CNC in the nanocomposites.

- In comparison with neat polyester, increases in the flexural strength, toughness, and total deflection were found for the nanocomposites. This is an indication of good interaction between the nanocrystals and the polyester, which was attained by addition of styrene to favor the CNC dispersion.
- Scanning electron microscopy fractography supports the evidence of improved CNC dispersion and interaction in association with surfaces roughness and undulation attributed to formation of microcracks surrounding the nanocrystals.
- Crystallinity index obtained by X-ray diffraction indicates that only 1 and 2 wt% of CNC promoted nucleation of atomically ordered regions. This suggested that nanocomposites with higher amounts of CNC, such as 3 wt%, might be associated with nanocrystals agglomeration that impairs the mechanical properties. Indeed, based on polynomial adjustment, an optimal CNC incorporation of 2 wt% is proposed for the nanocomposites.
- The resistance to thermal degradation of the nanocomposites is improved by 20 °C in comparison to the neat polyester. Walter absorption of the nanocomposites was found to be practically the same of the neat polyester. In principle this could also indicated a good dispersion, at least up to 2 wt%, and interaction of the highly hydrophilic CNC with the hydrophobic unsaturated polyester matrix.
- These results support potential future industrial application of CNC/polyester nanocomposites as ductile, lightweight and cost-effective substitute for conventional glass fiber/polyester composites.

## Figures and Tables

**Table 1:** Crystallinity index (CI) of polyester nanocomposites reinforced with different CNC contents.

CNC Content (wt%)	Crystalline Index, CI (%)
0 (neat polyester)	47.5 ± 1.4
1	50.3 ± 1.5
2	56.2 ± 1.7
3	44.9 ± 1.3

**Table 2:** Variance analysis of average flexural strength obtained for polyester matrix nanocomposites reinforced with 0 to 3 wt% CNC.

Variation Causes	DF	Sum of Squares	Mean Square	F (Calc.)	F Critical (Tab.)
Treatments	3	5200.11	1733.37	43.16	6.784.10^−8^
Residue	16	642.53	40.1582		
Total	19	5842.64			

**Table 3:** Results obtained from the Tukey’s pairwise comparisons (Q\p) between average values of flexural strength for polyester matrix nanocomposites reinforced with 0 to 3 wt% CNC.

CNC Content (wt%)	0	1	2	3
0	--	0.0001862	0.0001855	0.0001859
1	11.47	--	0.09536	0.9983
2	15.02	3.558	--	0.1289
3	11.71	0.2399	3.318	--

**Table 4:** Variance analysis of average elastic modulus obtained for polyester matrix nanocomposites reinforced with 0 to 3 wt% CNC.

Variation Causes	DF	Sum of Squares	Mean Square	F (Calc.)	F Critical (Tab)
Treatments	3	9.53019	3.17673	6.23	0.005248
Residue	16	8.15871	0.509919		
Total	19	17.6889			

**Table 5:** Results obtained from the Tukey’s pairwise comparisons (Q\p) between the average values of the elastic modulus for polyester matrix nanocomposites reinforced with 0 to 3 wt% CNC.

CNC Content (wt%)	0	1	2	3
0	--	0.009631	0.04202	0.009108
1	5.226	--	0.8784	1
2	4.175	1.05	--	0.8665
3	5.265	0.03953	1.09	--

**Table 6:** Variance analysis of average total deflection obtained for polyester matrix nanocomposites reinforced with 0 to 3 wt% CNC.

Variation Causes	DF	Sum of Squares	Mean Square	F (Calc.)	F Critical (Tab.)
Treatments	3	104.776	43.9254	21.79	6.777.10^−6^
Residue	16	25.6447	1.6028		
Total	19	130.421			

**Table 7:** Results obtained from the Tukey’s pairwise comparisons (Q\p) between the average values of the total deflection for polyester matrix nanocomposites reinforced with 0 to 3 wt% CNC.

CNC Content (wt%)	0	1	2	3
0	--	0.01182	0.0001972	0.0001956
1	5.081	--	0.021159	0.01889
2	9.736	4.655	--	0.999
3	9.831	4.75	0.09488	--

**Table 8:** Variance analysis of average flexural toughness obtained for polyester matrix nanocomposites reinforced with 0 to 3 wt% CNC.

Variation Causes	DF	Sum of Squares	Mean Square	F (Calc.)	F Critical (Tab.)
Treatments	3	1.49929	0.499764	22.06	6.265.10^−6^
Residue	16	0.362456	0.0226535		
Total	19	1.86175			

**Table 9:** Results obtained from the Tukey’s pairwise comparisons (Q\p) between the average values of the flexural toughness for polyester matrix nanocomposites reinforced with 0 to 3 wt% CNC.

CNC Content (wt%)	0	1	2	3
0	--	0.01403	0.0001888	0.0002243
1	4.96	--	0.006014	0.05368
2	10.52	5.559	--	0.6913
3	8.955	3.995	1.564	--
